# Lippes Loop intrauterine device left in the uterus for 50 years: case report

**DOI:** 10.1186/1472-6874-14-97

**Published:** 2014-08-15

**Authors:** Rosita Aniulienė, Povilas Aniulis

**Affiliations:** 1 Department obst/gyn of LUHS hospital, Eiveniu 2, Kaunas LT-50009, Lithuania; 2 Department of urology of LUHS hospital, Eiveniu 2, Kaunas LT-50009, Lithuania

**Keywords:** Intrauterine device (IUD), Contraception, LIPPES device

## Abstract

**Background:**

The first Lippes Loop intrauterine device (IUD) was introduced in 1962. It was a plastic double “S” loop, a trapezoid shaped IUD that closely fit around the contours of the uterine cavity, reducing the incidence of expulsion. This IUD was commonly used from the 1960’s to the 1980’s. Some authors state that the IUD can be left in the uterine cavity for an indefinite amount of time. Prolonged use of this device was common, however, it was associated with some complications like uterine bleeding during post-menopausal period and inflammatory pelvic diseases.

**Case presentation:**

The patient was a 74-years-old woman who was admitted to a university hospital due to urinary incontinence stress. The patient’s history included 2 deliveries and 20 years of menopause. During ultrasonography a normally sized and shaped uterus was found. The uterine cavity was expanded by 14 mm with some fluid. A “Lippes” loop was also seen in the uterine cavity. Both ovaries were atrophic without any abnormalities. The patient had her IUD inserted 50 years ago. Patient underwent TOT (tension obturator tape ) surgery for urinary incontinence. Evacuation of IUD and uterine curettage was also done.

**Conclusions:**

Fifty years of prolonged usage of LIPPES IUD had no influence on the woman’s health during our case.

## Background

The intrauterine device (IUD) is the most commonly used contraceptive method in the world [1-3]. It is effective, long-acting and rapidly reversible. It is one of the most cost-effective methods as well and can be used by most women, including those who have to avoid estrogens [2,3]. The use of the IUD is associated with some complications, including the risk of uterine perforation, malposition and expulsion of the device, abnormal bleeding and infections [3-6]. There have been reported cases of some women with expired IUD’s [4,6,7].

## Case presentation

**Figure 1:**
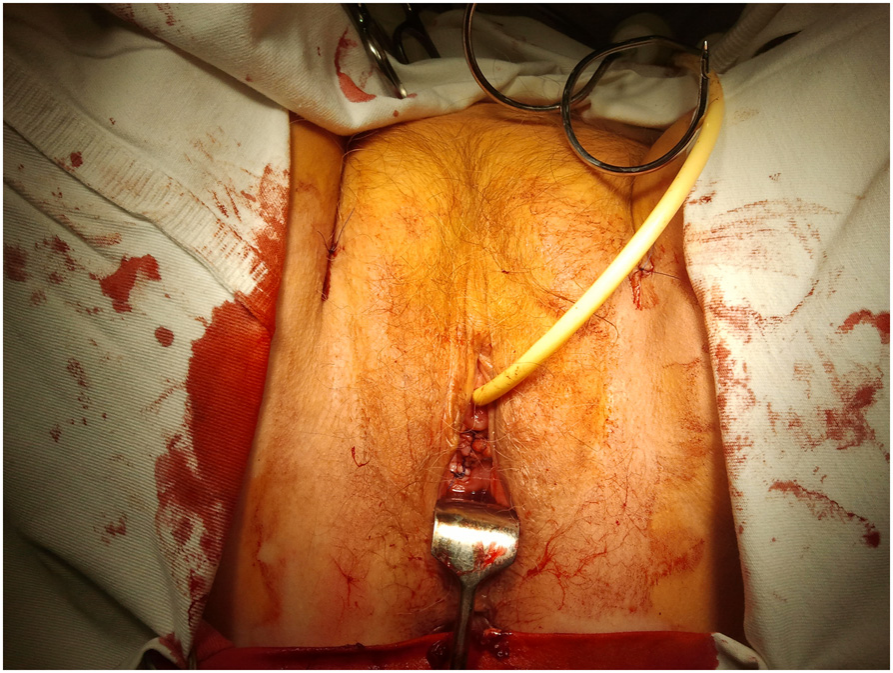
Patient during TOT operation.

**Figure 2:**
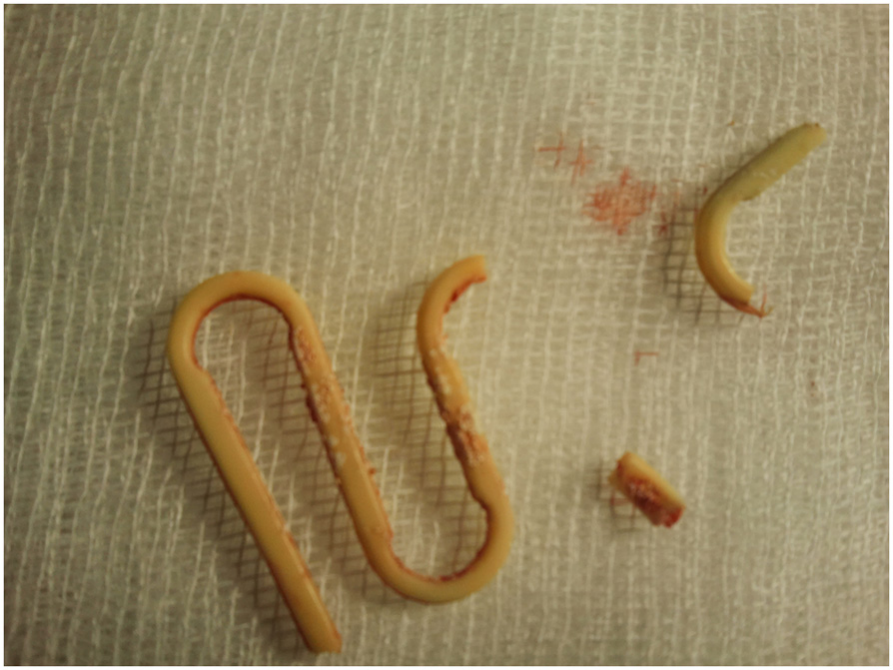
Lippes Loop IUD after evacuation.

We present a case of a intrauterine device that was inserted 50 years ago.The patient was a 74-years-old woman who was admitted to a university hospital due to stress urinary incontinence. The patient’s history included 2 deliveries and 20 years of menopause. During ultrasonography a normally sized and shaped uterus was found. The uterine cavity was expanded by 14 mm with some fluid. A “Lippes” loop was also found in the uterine cavity. Both ovaries were atrophic without any abnormalities. The patient had her IUD inserted 50 years ago. There had been attempts of extracting it after a few years of insertion, but was ultimately not successful and the woman forgot about it. The patient underwent TOT surgery for urinary incontinence. Evacuation of the IUD and uterine curettage was also done (Figures 1 and 2). Histological exam revealed no abnormalities. Postoperative period was without complications and two days after surgery the patient was discharged.

## Discussion

Lippes Loop intrauterine device was first introduced in 1962. It was a plastic double “S” loop, a trapezoidal shaped IUD that closely fit the contours of the uterine cavity, thereby reducing the incidence of expulsion [1,7]. This IUD was commonly used from the 1960s to the 1980s [1]. Some authors states that this IUD can be left in the uterine cavity for an indefinite amount of time [7]. Prolonged use of this device was common, however, it was associated with some complications like uterine bleeding during post-menopausal period and inflammatory pelvic disease [1,4-7].

There have been reported cases of prolonged usage of the IUD [1,4,8]. The time period of retained uterine devices varied from 22 to 44 years. Those women, most commonly, were presented to a specialist complaining of bleeding during a post-menopausal period [1,4]. The cause of the bleeding can be deducted to be the device’s migration into the uterine wall and also a chronic inflammatory response of the endometrium [4]. However, women with IUD presented with bleeding after a menopause should be closely examined, as it can represent a significant endometrial pathology. Investigation should include visualization of the endometrial cavity by hysteroscopy, as the measurement of the endometrial thickness during an ultrasound exam may be unreliable in the presence of an IUD [1]. Another reported case of a 32-year-old woman with a long-standing intrauterine device who developed an abdominal wall actinomycosis [8]. The diagnosis was established late by histopathological examinations after an initial surgical procedure was done, in which the abscess was evacuated and all the necrotic tissue was excised. Post operatively, the patient developed two intra-abdominal abscesses which were treated with surgical drainage. The combination of long-term high dose antibiotic therapy and surgery led to a successful management of the condition. Abdominal wall actinomycosis should be considered for intrauterine device users who are presented with abdominal abscesses of unknown origin [8].

Perforation of the uterus by an IUD has been reported on numerous occasions and, depending upon the degree of penetration through the myometrium, can be partial or complete [4]. A case was reported of a woman with Lippes loop who had a perforation of the uterus after having the device inserted 35 years ago. She presented an acute abdominal pain and underwent laparotomy and an postoperative pathological report demonstrated characteristics of actinomycosis associated with perforation [6]. It is noted that infection is most common during the first year after insertion of the device but may occur at any time. This is caused by microorganisms’ migration from the vagina and cervical canal along the threads of the uterine device [7]. To address these concerns, the authors (Farley TM, Rosenberg MJ et al.) have reviewed the World Health Organisation’s IUD clinical trial data to explore the incidence and patterns of the pelvic inflammatory disease (PID) risk with use of an IUD [9]. The overall rate of PID among 22.908 IUD insertions of a combined 51.399 woman years were about 1.6 cases per 1000 woman years of use. After adjustments of confounding factors, PID (pelvic inflammatory disease) risk was more than six times higher during the 20 days after insertion than during later times (unadjusted rates, 9.7 versus 1.4 per 1000 woman years, respectively) the risk was low and constant for up to eight years following up. Rates varied according to geographical area (highest in Africa and lowest in China) and were inversely associated with age. PID rates were lower among women who had IUDs inserted more recently [9].

In our case, the patient had no complains about the intrauterine device. She had never experienced bleeding episodes after a menopause and a pathohistological exam was clear of abnormal cells or infectious components in the curettage material.

Nowadays, intrauterine devices are one of the most frequently used reversible family planning methods in the world. The earlier IUD that was made of plastic materials has been replaced by new devices releasing copper or levonorgestrel. These modifications increased the already high efficiency [3,5]. The prospective cohort study was done in the US to analyze satisfaction and continuation of using different contraceptive methods. 12-month data from 5078 participants was analyzed. Continuation rates for long-acting reversible contraceptive methods ranged from 83 to 88% (women who were using IUD or the implant). The rates for other contraception forms (contraceptive pills and rings) ranged from 54 to 57%. It was concluded that long-acting reversible contraceptive methods are most effective and not user-dependent [2]. Many experts believe that IUD has highest continuation rates and the highest level of satisfaction amongst all the methods of contraception [2,3,5].

## Conclusions

Fifty years of prolonged usage of LIPPES IUD had no influence on women’s health in our case. For fifty years the patient was healthy, did not contract any diseases, PID or required surgery. IUD was found occasionally (the patient forgot about it) when the patient got admitted due to stress urinary incontinence.

## Consent

Informed consent from the patient was obtained for agreement to publish this manuscript (case report).

## Abbreviations

IUD: Intrauterine device, is used for the female contraception; TOT: Tension free transobturator tape, the operation is used for the treatment of stress urinary incontinence; PID: Pelvic inflammatory disease.

## Competing interest

None of the authors has a financial disclosure or competing interest.

## Authors’ contributions

RA surgeon of this case and general inspirator of main idea of the case report. PA participated in the design and coordination of the manuscript (case report). Both authors read and approved the final manuscript.

## Pre-publication history

The pre-publication history for this paper can be accessed here:

http://www.biomedcentral.com/1472-6874/14/97/prepub

## References

[B1] Pisal N Mammo M Case-series report: management of post-menopausal bleeding in the presence of an intrauterine device Contraception 2002 66 5 383 384 10.1016/S0010-7824(02)00364-5 12443971

[B2] Peipert J Zhao Q Allsworth J Petrosky E Madden T Esienberg D Secura G Continuation and satisfaction of reversible contraception Obstet Gynecol 2011 117 5 1105 1113 10.1097/AOG.0b013e31821188ad 21508749 PMC3548669

[B3] The ESHRE Capri Workshop Group Intrauterine devices and intrauterine systems Hum Reprod Update 2008 14 3 197 208 18400840 10.1093/humupd/dmn003

[B4] Ding D Hsu S Chu T Retained intrauterine device as an unusal cause of postmenopausal bleeding Tzu Chi Med J 2006 18 5 389 391

[B5] Wright EA Aisien OA Comparison of copper T-200 with Lippes Loop as a Contraceptive Device Int J Gynaecol Obstet 1989 29 2 173 177 10.1016/0020-7292(89)90849-7 2568294

[B6] Phupong V Sueblinvong T Pruksananonda K Taneepanichskul S Triratanachat S Uterine perforation with Lippes Loop Intrauterine Device-associated Actinomycosis: a case report and review of the literature Contraception 2000 61 5 347 350 10.1016/S0010-7824(00)00112-8 10906507

[B7] Pollock M Letting uterine device lie Br Med J 1982 285 395 396 10.1136/bmj.285.6339.395 6809099 PMC1499228

[B8] Lunca S Bouras G Romedea NS Pertea M Abdominal wall actinomycosis associated with prolonged use of an intrauterine device: a case report and review of the literature Int Surg 2005 90 4 236 240 16548322

[B9] Farley TM Rosenberg MJ Rowe PJ Chen JH Meirik O Intrauterine devices and pelvic inflammatory disease: an international perspective Lancet 1992 339 8796 785 788 10.1016/0140-6736(92)91904-M 1347812

